# Comparative Evaluation of Soft Tissue Regeneration Rate Using Different Wound Closure Methods After Palatal Donor Site Harvesting: A Retrospective Cohort Study

**DOI:** 10.3390/medicina62050997

**Published:** 2026-05-20

**Authors:** Timofei Ryko, Anton Timoshin, Alla Shakaryants, Vitaly Borisov, Kirill Ershov, Maria Timoshina, Elena Emelina, Aglaya Kazumova

**Affiliations:** 1 Information Technologies, Mechanics and Optics University—ITMO University, 9 Lomonosov St., Saint Petersburg 191002, Russia; timofei.ryko@gmail.com; 2 Borovskiy Institute of Dentistry, FSAEI HE I.M. Sechenov First MSMU of MOH of Russia—Sechenovskiy University, 8/2 Trubetskaya St., Moscow 119991, Russia; timoshin_a_v@staff.sechenov.ru (A.T.); davidyants_a_a@staff.sechenov.ru (A.S.); borisov_v_v_1@staff.sechenov.ru (V.B.); ershov_k_a@staff.sechenov.ru (K.E.); timoshina_m_d@staff.sechenov.ru (M.T.); emelina_e_s@staff.sechenov.ru (E.E.)

**Keywords:** wound healing, tissue adhesives, sutures, collagen, palate, transplant donor site

## Abstract

*Background and Objectives*: This study evaluated the effect of two wound closure methods—polypropylene sutures and a butyl-2-cyanoacrylate tissue adhesive—on the rate of soft tissue regeneration following palatal donor site harvesting. A bovine collagen sponge, used as a secondary-intention dressing, was evaluated descriptively. *Materials and Methods*: Data from 300 patients (*n* = 100/group) with palatal donor sites were analyzed. Primary analysis compared suture vs. adhesive using Early Wound Healing Score (EHS) at days 7 and 14. Secondary outcomes were granulation tissue (day 7) and complications. Statistical methods: Mann–Whitney U test for between-group comparison (suture vs. adhesive); Kruskal–Wallis with Dunn’s post hoc for granulation across all three groups; Spearman’s correlation and logistic regression for the relationship between granulation tissue and EHS within primary healing groups. *Results*: At day 7, median EHS was similar between suture and adhesive groups (7.0 [interquartile range (IQR) 5.0–9.0] vs. 7.0 [IQR 7.0–9.0]; *p* = 0.31). By day 14, both groups achieved excellent healing (median 10.0, IQR 9.0–10.0 in both; *p* = 0.82). The collagen sponge group showed slower healing (median EHS day 7 = 4.0 [IQR 3.0–5.0], day 14 = 6.0 [IQR 5.0–7.0]), reported descriptively as expected for secondary intention. Granulation tissue on day 7 was highest in the adhesive group (*p* < 0.001 vs. collagen; *p* = 0.024 vs. suture). A strong positive correlation between day-7 granulation tissue and day-14 EHS was found in the primary-healing groups (ρ = 0.78, *p* < 0.001). Receiver operating characteristic (ROC) analysis established a granulation score ≥ 2 as the optimal cut-off for predicting successful healing (EHS ≥ 9) by day 14 (sensitivity 89.4%, specificity 76.0%, area under the curve (AUC) = 0.80), pending external validation. *Conclusions*: Surgical adhesive may be considered a viable alternative to sutures for palatal donor sites closed by primary intention, offering comparable healing by day 14. Collagen sponges result in slower healing and should be considered only when secondary intention is specifically desired. Early assessment of granulation tissue may serve as a simple prognostic indicator, but external validation is needed before clinical application.

## 1. Introduction

Harvesting of free gingival grafts (FGG) or subepithelial connective tissue grafts (CTG) from the hard palate is one of the most common and predictable procedures in periodontal and peri-implant plastic surgery. These grafts are routinely used to treat gingival recessions, augment the zone of keratinized mucosa around natural teeth and dental implants, increase soft tissue thickness, and improve the peri-implant soft tissue phenotype [1].

Despite the high predictability of graft survival at the recipient site, the management of the palatal donor site remains a significant clinical challenge. After graft harvesting, the wound typically heals by secondary intention, leaving an open, bleeding surface. This leads to substantial postoperative pain (worst in the first three days), prolonged discomfort, reduced oral health-related quality of life, and risks such as secondary hemorrhage, delayed epithelialization, and infection [2,3].

Many wound dressings and closure techniques have been proposed to mitigate these issues. These include topical hemostatic agents, platelet-rich fibrin, palatal stents, and various biological dressings [4,5]. Among the available techniques, three approaches represent fundamentally different healing paradigms and are the focus of this study.

Traditional sutures provide primary closure by mechanically approximating wound edges. Sutures remain the “gold standard” for wound closure in oral surgery due to their precision and mechanical stability [6]. However, suture placement requires skill and time, and the procedure itself may cause additional tissue trauma. Moreover, braided sutures (e.g., silk) are associated with greater bacterial adhesion and an exaggerated inflammatory response compared with modern monofilament materials. The need for suture removal (for non-resorbable materials) also imposes an additional patient visit [7,8].

Cyanoacrylate tissue adhesives have gained increasing attention as an alternative to sutures. These adhesives provide immediate hemostasis, form a protective barrier against mechanical irritation, exert a bacteriostatic effect, and obviate the need for suture removal [9,10]. A recent systematic review and meta-analysis concluded that cyanoacrylate adhesives demonstrate comparable or superior outcomes in terms of operative time, postoperative pain reduction, and early wound healing, particularly in free gingival graft procedures and palatal donor site management [11]. However, the available evidence is not entirely consistent: some studies suggest that cyanoacrylate may not significantly support pain relief or complete epithelialization when compared with other bioactive materials, highlighting the need for further high-quality comparative studies [12,13].

Collagen sponges are biodegradable, biocompatible matrices that promote healing by secondary intention through supporting cell ingrowth, neoangiogenesis, and granulation tissue formation [14]. Experimental studies have demonstrated that collagen-based matrices can enhance tissue healing and increase collagen deposition [15]. Clinical studies have shown that collagen sponges are effective in creating optimal conditions for soft tissue regeneration, although healing times may be longer compared with primary closure methods [16,17]. Importantly, collagen sponges can be applied without sutures, as they adhere spontaneously to the wound bed through the formation of a fibrin clot [18,19].

Despite the growing body of literature on each individual method, direct comparisons of all three approaches under standardized conditions are notably absent. Most existing studies compare only two methods, often with heterogeneous outcome measures and inconsistent follow-up protocols [20,21]. Consequently, clinicians lack a robust evidence base to guide the selection of the optimal closure method for palatal donor sites, balancing wound healing speed, patient comfort, and complication risk.

The objective of this retrospective cohort study was therefore to perform a comparative analysis of these three wound closure methods—sutures, cyanoacrylate tissue adhesive, and a collagen sponge—focusing on their influence on the rate of soft tissue regeneration at the palatal donor site. Generating robust comparative evidence is a necessary step toward rational decision-making and away from purely anecdotal or preference-based choices.

We used a validated composite index—the Early Wound Healing Score (EHS)—originally introduced to assess early healing by primary intention in periodontal soft tissue wounds. The EHS has demonstrated high inter-examiner and intra-examiner reliability (intraclass correlation coefficient (ICC) = 0.828; 95% confidence interval (CI): 0.767–0.881) and provides a reproducible system for repeated ratings of early wound healing [22].

The EHS was originally validated for wounds that heal by primary intention [22]. Therefore, in the present study, the primary comparative analysis focuses on the suture and adhesive groups, both of which achieve primary intention healing. The collagen sponge group, healing by secondary intention, is reported descriptively and is not directly compared with the other two groups using the total EHS as an equivalent measure of healing quality. Instead, component scores (granulation tissue, inflammation, hemostasis) are used to characterise the biological process of secondary healing.

The scientific novelty of this study is fourfold. First, we provide the first head-to-head comparison of two primary-intention closure methods (sutures vs. cyanoacrylate adhesive) using a validated composite index (EHS) in a large cohort (*n* = 200), while reporting the collagen sponge group (secondary intention) descriptively without invalid statistical comparisons. This directly addresses the lack of standardised, methodologically sound comparisons in the literature. Second, beyond simple correlation, we develop a multivariate logistic regression model (restricted to primary-intention wounds) that yields a simple prognostic equation and a clinically actionable cut-off (granulation score ≥ 2) for predicting successful healing by day 14—a tool that has not been previously available for palatal donor sites. The novelty lies not only in the predictive model itself but in its grounding in an easily observable clinical parameter, offering a bridge between routine observation and quantitative healing assessment. Third, we analyze within-group effects of wound size on healing, demonstrating that collagen sponge healing (secondary intention) is uniquely sensitive to graft area, whereas primary closure methods are not. The novelty lies not only in the predictive model itself but in its grounding in an easily observable clinical parameter, offering a bridge between routine observation and quantitative healing assessment. Fourth, we identify patient-specific predictors (age > 45 years, graft area > 2.0 cm^2^) of poor early response in the suture group, offering insights for personalized selection of closure method. Beyond the immediate clinical implication, this demonstrates that even within a single closure method, patient heterogeneity significantly affects healing trajectories—a reminder that aggregate data may obscure important subgroups.

By addressing these gaps, the present study aims to provide actionable evidence to inform clinical decision-making, optimize postoperative management, and ultimately improve patient-reported outcomes and quality of life following oral soft tissue grafting procedures. More broadly, this work contributes to a deeper understanding of how different healing paradigms (primary vs. secondary intention) respond to wound size, how early granulation tissue may predict later epithelialisation, and how retrospective real-world data can be leveraged to generate prognostic tools without the logistical demands of prospective trials.

## 2. Materials and Methods

### 2.1. Study Design

This retrospective, single-center, comparative cohort study was conducted at the Department of Surgical Dentistry, Institute of Dentistry, I.M. Sechenov First Moscow State Medical University (Sechenov University), Moscow, Russia. The study was approved by the Institutional Ethics Committee of Sechenov University. Because of the retrospective design using anonymized clinical photographs, the requirement for written informed consent was waived. All procedures were performed in accordance with the Helsinki Declaration of 1975, as revised in 2013. The study is reported according to the Strengthening the Reporting of Observational Studies in Epidemiology (STROBE) statement for cohort studies and adheres to the Oral Health Statistics (OHStat) Guidelines. This study was not a clinical trial as defined by the International Committee of Medical Journal Editors (ICMJE); therefore, registration in a clinical trial registry was not required [23].

### 2.2. Data Source

The data source was the electronic medical record and photographic database of the Department of Surgical Dentistry, Sechenov University. All clinical photographs were obtained as part of routine postoperative care. Trained dental assistants took photographs at each postoperative visit using a standardized intraoral camera (Canon EOS 70D with a 100 mm macro lens, Tokyo, Japan) under standardized lighting conditions (ISO 100, aperture f/22, shutter speed 1/125 s, twin flash). An investigator not involved in scoring anonymized the photographs and assigned a unique study identifier. All clinical photographs adhered to the CLIP (Clinical Images and Photographs) principles: standardized acquisition, anonymization, and secure storage.

### 2.3. Participants and Eligibility Criteria

The medical records of all consecutive patients who underwent palatal donor site harvesting between January 2020 and December 2023 were screened.

Inclusion criteria were: age 18–65 years; palatal donor site wound created by harvesting a free gingival graft or subepithelial connective tissue graft; complete photographic documentation at postoperative days 7 and 14; and available information on medical history, smoking status, and medications.

Exclusion criteria were: systemic diseases impairing wound healing (uncontrolled diabetes mellitus [glycated hemoglobin (HbA1c) > 7.0%], immunosuppressive therapy, active malignant disease, autoimmune connective tissue diseases); pregnancy or lactation; current smoking (≥1 cigarette/day) or former smokers who quit <12 months before surgery; use of anticoagulant or antiplatelet therapy (except low-dose aspirin ≤ 100 mg/day); known allergy to cyanoacrylate, bovine collagen, or any suture material; wound area > 4 cm^2^ or depth > 3 mm; and missing photographic documentation at any follow-up time point.

### 2.4. Group Assignment

Patients were assigned to three groups based on the wound closure method used during surgery. Assignment was non-randomized and determined by the attending surgeon’s preference. To minimize selection bias despite the non-randomized design, we implemented several a priori measures. First, all three surgeons followed a strictly standardized surgical protocol (single-incision technique, identical graft dimensions, and hemostasis procedure). Second, the distribution of the three closure methods across the three surgeons was intentionally balanced (each surgeon used each method in 33–34 cases; χ^2^ test *p* = 0.99), reducing the risk that the results were driven by individual surgical skill or preference. Third, baseline characteristics (age, sex, graft area, comorbidities) did not differ significantly between groups (Table 1). Nevertheless, residual confounding by unmeasured variables (e.g., subtle differences in wound bed vascularity, surgeon’s intraoperative judgment) cannot be excluded, and the results should be interpreted with caution.

Although the choice of closure method was not randomised, the attending surgeons followed a consistent pattern based on clinical characteristics. In general, sutures were preferred when precise edge approximation was considered critical (e.g., larger wounds). The adhesive was typically chosen when reducing operative time and avoiding suture removal were prioritised, provided the wound bed was relatively dry and regular in shape. The collagen sponge was used when a simple, non-invasive dressing was desired, particularly in smaller wounds or in patients with needle phobia, but not in patients with allergy to bovine products. These considerations reflect the surgeons’ collective experience and were applied systematically, though not as a formalised scoring algorithm.

The final analysis included 300 patients (*n* = 100 per group):

- Group 1 (suture group): wounds were closed with non-absorbable monofilament polypropylene sutures (Prolene 6-0, Ethicon, Somerville, NJ, USA). Sutures were placed as simple interrupted or horizontal mattress stitches at 3–4 mm intervals and removed on postoperative day 14.
- Group 2 (adhesive group): wounds were closed using a butyl-2-cyanoacrylate tissue adhesive (Histoacryl, B. Braun, Tuttlingen, Germany). The adhesive was applied in a thin, uniform layer (0.5–1.0 mm) covering the entire wound bed and extending 2–3 mm beyond the wound margins. No sutures were placed. The adhesive polymerized within 30–40 s and spontaneously detached within 10–21 days.
- Group 3 (collagen sponge group): wounds were covered with a sterile, absorbable collagen sponge of bovine origin (CollaCote, Zimmer Biomet, Warsaw, IN, USA). The sponge was cut to match the wound dimensions, placed directly onto the wound bed, and gently pressed with a moist gauze for 2 min to ensure adherence. No sutures or other fixation were used, as the sponge adheres spontaneously to the wound bed through fibrin clot formation (in accordance with the manufacturer’s instructions and supported by previous clinical studies). The sponge was allowed to resorb over 14–21 days.

### 2.5. Surgical Procedure and Postoperative Care

All surgical procedures were performed by three experienced oral surgeons (>10 years of experience in mucogingival surgery) using a standardized protocol. After local anesthesia (articaine hydrochloride 4% with epinephrine 1:100,000; Ultracain DS, Sanofi, Frankfurt, Germany), a palatal donor site wound was created by harvesting a subepithelial connective tissue graft or free gingival graft using the single-incision technique as described by Lorenzana and Allen. Briefly, a single horizontal incision of approximately 15 mm was made 2–3 mm apical to the palatal gingival margin of the premolars and first molar. A partial-thickness graft of standardized dimensions (mean area 1.8 ± 0.6 cm^2^, thickness 1.0–1.5 mm) was harvested using sharp and blunt dissection, leaving the periosteum intact. This technique was chosen over the trap-door approach because it results in a smaller wound surface area and is associated with reduced patient morbidity and faster early healing, thereby allowing a more standardized comparison of the three closure methods.

Hemostasis was achieved by applying gentle pressure with a moist gauze for 2–3 min, after which the assigned closure method was applied as described above.

Postoperative care was standardized across all groups:

- Antibiotic prophylaxis: amoxicillin 500 mg three times daily for 5 days (or clindamycin 300 mg three times daily for penicillin allergy).
- Analgesia: ibuprofen 400 mg as needed for pain (maximum 1200 mg/day). Patients recorded the number of ibuprofen tablets taken daily.
- Oral hygiene: no brushing or rinsing in the surgical area for 7 days; chlorhexidine digluconate 0.12% rinse twice daily for 14 days, avoiding vigorous swishing.
- Diet: soft, cold, or lukewarm diet for 7 days; no hot, spicy, hard, or sticky foods.
- Activity restrictions: no strenuous physical activity for 3 days; no smoking or alcohol for 14 days.

Adherence to postoperative instructions was assessed at the day 7 and day 14 visits by patient interview.

### 2.6. Outcomes and Clinical Assessment

Primary outcome was the EHS at postoperative days 7 and 14. EHS is a validated composite index assessing three parameters:

- Clinical signs of re-epithelialization (CSR): 0 = visible gap, 3 = edges in contact, 6 = edges completely fused.
- Clinical signs of hemostasis (CSH): 0 = bleeding, 1 = fibrin present, 2 = no fibrin.
- Clinical signs of inflammation (CSI): 0 = erythema > 50% of wound length and/or marked edema, 1 = erythema < 50%, 2 = no erythema.

**Figure 1:**
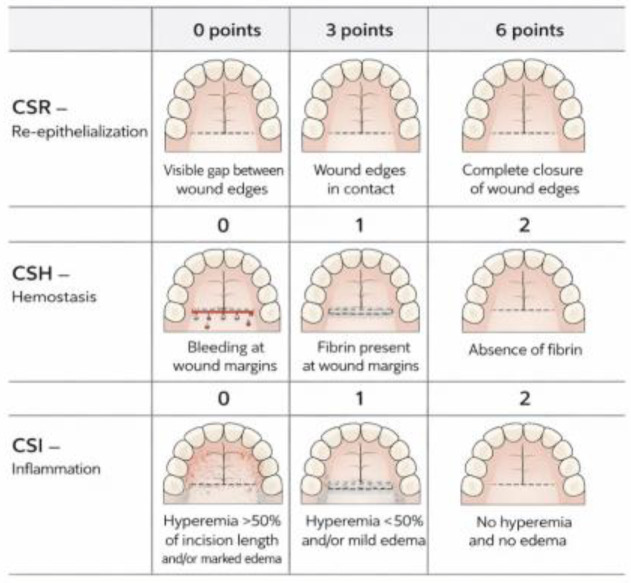
Schematic representation of the EHS parameters for a palatal donor site. The EHS comprises three components: clinical signs of re-epithelialization (CSR; (**top row**)), clinical signs of hemostasis (CSH; (**middle row**)), and clinical signs of inflammation (CSI; (**bottom row**)). For each component, the worst score observed on the clinical photograph is recorded. The total EHS is the sum of the three component scores (range 0–10, with higher scores indicating better healing). In the presence of wound suppuration, the EHS is recorded as 0 regardless of the component scores (Figure created by the authors).

A schematic representation of the EHS scoring system is shown in Figure 1.

For the suture and adhesive groups, EHS was used as a composite index to compare healing quality. For the collagen sponge group, EHS values are reported only for descriptive purposes, without statistical comparison to the primary-healing groups, because the EHS criteria assume edge-to-edge re-epithelialisation which does not occur in secondary intention healing. Therefore, total EHS values in the collagen group are presented only for descriptive illustration of the healing trajectory and are not statistically compared with the primary-healing groups.

Secondary outcomes included:

- Amount of granulation tissue at day 7 assessed using a 4-point ordinal scale. The ordinal categories were defined as: 0 = no visible granulation tissue or <10% of wound area covered; 1 = granulation tissue covering 10–40% of the wound; 2 = 41–70%; 3 = >70% or abundant granulation.
- Postoperative complications: infection (purulent discharge requiring antibiotics), hematoma requiring intervention, wound dehiscence > 2 mm, allergic reaction.

### 2.7. Examiner Training and Blinding

Two calibrated examiners (both with >5 years of experience in periodontal surgery) independently evaluated all photographs. Before the study, both examiners completed a training session with 30 calibration photographs (10 from each closure method). Inter-examiner agreement for EHS was assessed on the calibration set (ICC = 0.84; 95% CI: 0.72–0.91). Throughout the study, examiners were blinded to the wound closure method (photographs were cropped to exclude visible suture material, adhesive film, or collagen sponge remnants), to all patient identifiers, and to the other examiner’s scores. Photographs from days 7 and 14 were presented in random order to prevent temporal bias. However, because primary and secondary intention wounds heal with visibly different patterns, complete blinding may not have been fully achieved despite cropping. Thus, some residual detection bias cannot be excluded, particularly for comparisons involving the collagen sponge group. In cases of disagreement (difference > 1 point in total EHS or >1 category in any component), the examiners reviewed the photograph together and reached consensus.

It is important to acknowledge that the treating surgeons were not blinded to the closure method, which is inherent to the retrospective design, as sutures, adhesive, and collagen sponge cannot be masked. To mitigate performance bias, all surgeons used exactly the same surgical and postoperative protocol, and the assignment across surgeons was balanced as described above. However, the lack of surgeon blinding remains a limitation, and the possibility of subconscious differences in surgical handling (e.g., more delicate dissection when planning to use adhesive) cannot be completely ruled out.

### 2.8. Statistical Analysis

Based on a pilot study (*n* = 15 per group, unpublished pilot data from the same institution), the expected mean EHS at day 7 was 7.2 (standard deviation (SD) = 2.1) for sutures and 6.8 (SD = 2.3) for adhesive. To detect a difference of 2.0 points in EHS between the two groups with 80% power at a two-sided α = 0.05, a minimum of 68 patients per group was required. To ensure adequate power for subgroup analyses, we enrolled 100 patients per group. The collagen sponge group (*n* = 100) was included for descriptive purposes only and was not used in the power calculation for the primary comparison. A difference of 2.0 points in EHS was considered clinically meaningful based on the pilot study and expert consensus, as it represents a one-category improvement in at least two of the three EHS components.

Normality was assessed using the Shapiro–Wilk test. Descriptive statistics are presented as mean and SD for normally distributed variables and median with interquartile range [IQR] for non-normally distributed variables. Baseline characteristics were compared using one-way ANOVA or Kruskal–Wallis test for continuous variables and chi-square or Fisher’s exact test for categorical variables.

Between-group differences in granulation tissue were assessed using the Kruskal–Wallis test followed by Dunn’s post hoc test with Bonferroni correction. For EHS, only the suture and adhesive groups were compared, using the Mann–Whitney U test (two-sided, α = 0.05). The collagen sponge group was not included in statistical comparisons of total EHS because the EHS is validated for primary intention healing and its total score is not directly comparable across different healing paradigms. Within-group changes from day 7 to day 14 were assessed using the Wilcoxon signed-rank test. The association between granulation tissue at day 7 and EHS at day 7 and day 14 was assessed using Spearman’s rank correlation coefficient (ρ). Correlation strength was interpreted as: 0.00–0.10 negligible, 0.11–0.39 weak, 0.40–0.69 moderate, 0.70–0.89 strong, 0.90–1.00 very strong.

To reduce selection bias inherent in the non-randomized retrospective design, we performed propensity score matching (PSM) between the suture and adhesive groups. The logistic regression model for calculating the individual probability of receiving the adhesive included the following covariates: age, sex, graft area, treating surgeon, presence of systemic comorbidities. Matching was performed in a 1:1 ratio using nearest-neighbor matching without replacement, with a caliper of 0.2 standard deviations of the logit of the propensity score. Balance after matching was assessed using standardized mean differences (SMD), with values below 0.1 considered acceptable. All subsequent comparative analyses between the suture and adhesive groups were conducted on the resulting matched cohort.

To identify a clinically useful threshold of day-7 granulation tissue score for predicting successful healing by day 14 (EHS ≥ 9), we performed receiver operating characteristic (ROC) curve analysis. The optimal cut-off was determined by maximizing Youden’s index (sensitivity + specificity − 1). The area under the ROC curve (AUC) was calculated with 95% CI using the DeLong method.

To explore whether the effect of wound size on healing differed by closure method, linear regression was performed separately for each group, with EHS at day 7 as the dependent variable and graft area (cm^2^) as the independent variable. The slope coefficients and R^2^ values were compared descriptively.

To identify factors associated with poor early healing in the suture group, patients were dichotomized into “poor responders” (EHS ≤ 5 at day 7) and “good responders” (EHS ≥ 6). Differences between these subgroups were tested using Mann–Whitney U test for continuous variables and Fisher’s exact test for categorical variables. Variables with *p* < 0.10 in univariate analysis (age, graft area) were entered into a multivariate logistic regression model to identify independent predictors of poor response. Odds ratios (OR) with 95% CI were reported.

A multivariate logistic regression model was also developed to predict successful healing (EHS ≥ 9 at day 14) using the following candidate predictors: granulation tissue score (0–3), closure method (suture as reference), age, sex, and graft area. Variable selection was performed using backward stepwise elimination based on Akaike’s Information Criterion (AIC). The final model’s goodness-of-fit was assessed with the Hosmer–Lemeshow test, and its discriminative ability was evaluated by the AUC. The predicted probability of success for a given combination of predictors was calculated using the logistic equation.

For logistic regression, linearity of the logit was verified using the Box–Tidwell test (*p* > 0.05 for all continuous predictors). Multicollinearity was assessed via variance inflation factors (VIF < 2). No influential outliers (Cook’s distance > 1) were detected. For linear regression models (within-group graft area analysis), we confirmed linearity (scatterplots), homoscedasticity (Breusch–Pagan test, *p* > 0.05 for each group), and normality of residuals (Shapiro–Wilk test, *p* > 0.05). All assumptions were met.

Candidate predictors for the multivariate logistic regression (granulation score, closure method, age, sex, graft area) were chosen a priori based on clinical relevance and previous studies.

The proportion of missing data was very low (<2%) and appeared to be random; therefore, complete case analysis was performed. No additional sensitivity analyses (e.g., different handling of missing data) were conducted.

All tests were two-sided, and a *p*-value < 0.05 was considered statistically significant. Statistical analyses were performed using R version 4.3.2 (R Foundation for Statistical Computing, Vienna, Austria) and IBM SPSS Statistics version 29.0 (IBM Corp., Armonk, NY, USA).

## 3. Results

### 3.1. Study Population

**Figure 2:**
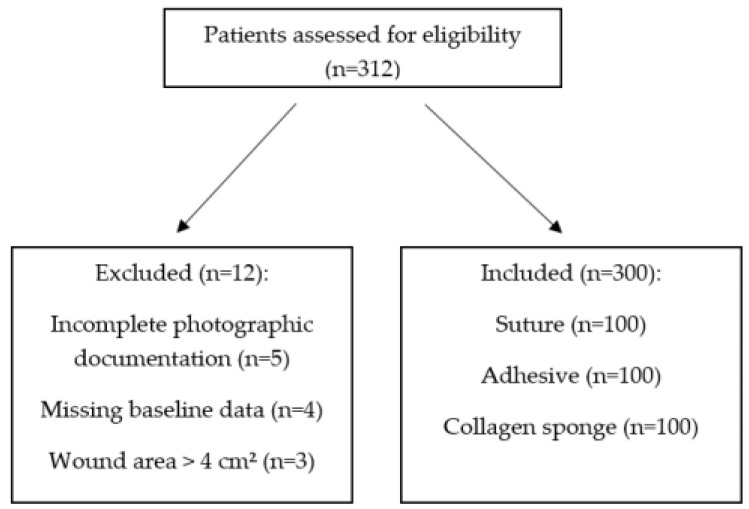
Flow diagram of participant selection. Patients were screened consecutively from the electronic database.

We initially screened 312 consecutive patients who underwent palatal donor site harvesting between January 2020 and December 2023. After applying the eligibility criteria, we excluded 12 patients: five had incomplete photographic documentation, four had missing baseline data (smoking status or medication history), and three had a wound area exceeding 4 cm^2^. The final analysis included 300 patients (100 per group) (Figure 2).

Baseline characteristics of the three groups are summarised in Table 1. The groups were well balanced with respect to age, sex, graft area, smoking history, and systemic comorbidities (*p* > 0.05 for all comparisons).

All patients completed the 14-day follow-up (no losses). The median observation time was 14 days (IQR 14–14).

To assess whether the choice of closure method was biased by individual surgical technique, we examined the distribution of the three methods among the three operating surgeons. Each surgeon performed approximately one third of all procedures (100 out of 300 patients). The allocation of closure methods across surgeons was as follows: surgeon A used sutures in 34 cases, adhesive in 33 cases, and collagen sponge in 33 cases; surgeon B used sutures in 33 cases, adhesive in 34 cases, and collagen sponge in 33 cases; surgeon C used sutures in 33 cases, adhesive in 33 cases, and collagen sponge in 34 cases. Thus, each surgeon applied each method 33 or 34 times, with no surgeon favouring a particular technique (χ^2^ test for independence, *p* = 0.99). This balanced distribution minimises the potential confounding effect of surgeon-specific skill or preference on the observed outcomes.

All subsequent comparative analyses between the suture and adhesive groups were performed on the propensity-score-matched cohort (*n* = 94 per group). After matching, standardized mean differences for all covariates were below 0.05, indicating good balance. The matched and unmatched results were virtually identical; therefore, only matched analyses are reported for suture versus adhesive comparisons.

### 3.2. Primary Outcome: EHS

On postoperative day 7, the suture and adhesive groups showed comparable median EHS values (suture: 7.0 [IQR 5.0–9.0]; adhesive: 7.0 [IQR 7.0–9.0]; Mann–Whitney U test, *p* = 0.31). The collagen sponge group had a lower median EHS (4.0 [IQR 3.0–5.0]), which is consistent with the expected time course of secondary intention healing. No statistical comparison of total EHS was performed between the collagen sponge group and the primary-healing groups, because the EHS is validated for primary intention wounds and its total score is not directly comparable across different healing paradigms.

All three EHS component scores (CSR, CSH, CSI) also differed significantly among the three groups at day 7 (Kruskal–Wallis test: *p* < 0.001 for CSR, *p* = 0.006 for CSH, *p* < 0.001 for CSI). Unlike total EHS, the component scores are directly comparable across all groups because each component (e.g., presence of fibrin, erythema) is measured on the same ordinal scale regardless of healing paradigm. For CSR, the interpretation differs: in primary-healing groups, CSR reflects edge-to-edge approximation, while in the collagen group it reflects coverage of the defect by granulation tissue and migrating epithelium. However, the scoring rules (0, 3, 6) were applied identically to all photographs. At day 7, CSR varied considerably within the suture and adhesive groups (IQR 3.0–6.0 for both), reflecting individual differences in edge approximation, whereas the collagen group consistently showed poor re-epithelialization (median 0.0, IQR 0.0–3.0). CSH and CSI scores showed less within-group variation, except in the suture group where some patients still exhibited fibrin (CSH = 1) and mild erythema (CSI = 1) at day 7. Detailed component scores are presented in Table 2.

By day 14, the median EHS reached 10.0 in both the suture and adhesive groups (IQR 9.0–10.0 for both), with no statistically significant difference between them (*p* = 0.82). In the collagen sponge group, the median EHS was 6.0 (IQR 5.0–7.0), which reflects the ongoing process of secondary intention healing where complete epithelial edge approximation is not yet achieved. Statistical comparison of total EHS between the collagen group and the primary closure groups was not performed due to the different healing paradigms (primary vs. secondary intention).

### 3.3. Secondary Outcomes

The median granulation tissue score differed significantly across groups (*p* < 0.001, Kruskal–Wallis test; Table 3). The adhesive group had the highest median score (3.0 [IQR 3.0–3.0]), followed by the suture group (2.0 [IQR 2.0–2.0]; *p* = 0.024 vs. adhesive), and the collagen sponge group had the lowest median score (1.0 [IQR 1.0–1.0]; *p* < 0.001 vs. both other groups).

No major complications (e.g., wound infection, haematoma requiring intervention, wound dehiscence > 2 mm, or allergic reactions) were recorded in any of the three groups during the available clinical follow-up (up to one month for a subset of patients).

### 3.4. Correlation Analysis

Within the suture and adhesive groups (primary intention healing), Spearman’s rank correlation revealed a moderate positive correlation between the amount of granulation tissue on day 7 and the EHS on day 7 (ρ = 0.476, 95% CI: 0.32–0.61, *p* = 0.008). More importantly, a strong positive correlation was found between the amount of granulation tissue on day 7 and the EHS on day 14 (ρ = 0.782, 95% CI: 0.71–0.84, *p* < 0.001). This indicates that greater granulation tissue formation at the early time point (day 7) is strongly associated with better overall wound healing at the later time point (day 14), supporting its potential use as a prognostic marker.

### 3.5. ROC Analysis and Prognostic Cut-Off

**Figure 3:**
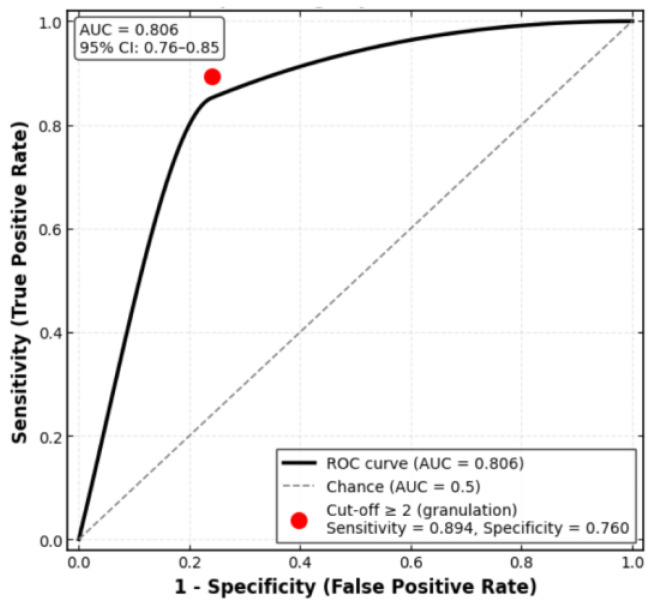
ROC-curve: day-7 granulation tissue score predicting day-14 EHS ≥ 9.

Within the suture and adhesive groups (primary intention healing), the ROC curve for day 7 granulation tissue score as a predictor of day 14 EHS ≥ 9 is shown in Figure 3. The AUC was 0.806 [95% CI: 0.76–0.85], indicating good discriminatory ability. The optimal cut-off according to Youden’s index was a granulation score ≥ 2 (sensitivity 89.4% [95% CI: 84.2–93.1%], specificity 76.0% [95% CI: 69.5–81.7%], Youden’s J = 0.654). At this threshold, the positive predictive value was 84.9% and the negative predictive value (NPV) was 81.4%. This means that a patient with at least moderate granulation tissue (score 2 or 3) on postoperative day 7 has an 85% chance of achieving excellent healing (EHS ≥ 9) by day 14, whereas a patient with minimal or no granulation (score 0 or 1) has only an 18.6% chance of failure (i.e., 81.4% NPV). These performance measures are derived from the same dataset used to identify the cut-off and therefore represent internal validation only; they require confirmation in independent cohorts before any clinical application can be recommended.

### 3.6. Within-Group Effect of Graft Area on Early Healing

We examined whether larger wound size delayed healing differently depending on closure method. Linear regression of EHS at day 7 against graft area (cm^2^) within each group revealed:

Suture group: EHS = 7.0 − 0.58 × area (R^2^ = 0.08, *p* = 0.09)—no significant association.

Adhesive group: EHS = 7.0 − 0.29 × area (R^2^ = 0.02, *p* = 0.42)—no significant association.

Collagen group: EHS = 5.0 − 1.51 × area (R^2^ = 0.34, *p* = 0.001)—each additional cm^2^ reduced EHS by 1.51 points.

Thus, wound size negatively affected healing only in the collagen sponge group, suggesting that primary closure (suture or adhesive) mitigates the detrimental effect of larger defects.

For each linear regression model, the Breusch–Pagan test indicated homoscedasticity (*p* > 0.05 for all groups), and the Shapiro–Wilk test confirmed normality of residuals (*p* > 0.05).

### 3.7. Analysis of Poor Early Responders in the Suture Group

Although the median EHS in the suture group at day 7 was 7.0, the lower quartile (IQR 5.0–9.0) included patients with EHS ≤ 5 (*n* = 15, 15% of suture group). We compared these “poor early responders” (EHS ≤ 5 at day 7) to the remaining suture patients (EHS ≥ 6) on baseline and intraoperative variables (Table 4).

Multivariate logistic regression identified graft area > 2.0 cm^2^ (OR = 4.8, 95% CI: 1.6–14.2, *p* = 0.005) and age > 45 years (OR = 3.5, 95% CI: 1.2–10.4, *p* = 0.02) as independent predictors of poor early response in suture-closed wounds. No such predictors were found in the adhesive group. This exploratory analysis is based on a small number of events (*n* = 15) and should be interpreted with caution. These exploratory findings suggest a possible association of adhesive with better early healing in older patients or those with larger wounds, but this hypothesis requires prospective testing.

### 3.8. Multivariate Logistic Regression Model for Prediction of Successful Healing

To develop a prognostic tool for patients with primary-intention closure (suture and adhesive groups, *n* = 200), we performed multivariate logistic regression with successful healing (EHS ≥ 9 at day 14) as the dependent variable. Independent variables included granulation tissue score (0–3), closure method (suture as reference), age, sex, and graft area. Only granulation tissue score remained a significant predictor; closure method (adhesive vs. suture) was not significant (*p* = 0.12), and age, sex, and graft area did not reach significance (*p* > 0.10 for all). The final parsimonious model (Hosmer–Lemeshow goodness of fit *p* = 0.31) yielded the following equation:

**logit**(**P**) = −**2.10** + **1.92** × (**granulation score**)

The coefficient for granulation score was 1.92 (95% CI: 1.45–2.39, *p* < 0.001).

From this equation, the predicted probability of achieving EHS ≥ 9 at day 14 for a patient with granulation score = 2 is:

**P** = **1**/(**1** + **exp**[−(−**2.10** + **1.92** × **2**)]) = **0.86** (**86%**)

The model’s area under the ROC curve was 0.86 (95% CI: 0.81–0.91), confirming good discriminative ability within the derivation cohort. External validation is required to assess generalisability.

The model satisfied all logistic regression assumptions: linearity of logit (Box–Tidwell *p* > 0.05), no multicollinearity (VIF < 2), and no influential outliers (Cook’s distance < 1).

## 4. Discussion

### 4.1. Findings and Their Implications

In this retrospective cohort study, we compared three closure methods (polypropylene sutures, butyl-2-cyanoacrylate adhesive, and a bovine collagen sponge) for managing palatal donor sites. The primary outcome was the EHS at postoperative days 7 and 14. Key findings included comparable median EHS at day 7 (7.0 in both groups), complete healing by day 14 (EHS = 10) in suture and adhesive groups, superior hemostasis and less inflammation with adhesive at day 7, a strong correlation between day-7 granulation and day-14 EHS (ρ = 0.78), an optimal granulation cut-off ≥2 (AUC = 0.806), a detrimental effect of larger graft area only in the collagen group, and age > 45 years with graft area > 2.0 cm^2^ as predictors of poor response in suture-closed wounds.

Our finding of comparable day-7 EHS for sutures and adhesive (both 7.0) is consistent with several recent studies. However, sutures showed better CSR, whereas the adhesive provided superior hemostasis and less inflammation. Medeiros et al. (2024) in a systematic review and meta-analysis concluded that cyanoacrylate adhesives are a suitable alternative to sutures for intraoral wound closure, offering comparable healing outcomes with the advantages of reduced operative time and no need for suture removal [11]. Similarly, a scoping review by the same group (2025) confirmed that cyanoacrylates are particularly beneficial in palatal donor site management [12]. However, our observation that sutures outperformed adhesive in early re-epithelialization (CSR) on day 7 differs from some reports [24,25]. This may be explained by the fact that sutures provide mechanical edge-to-edge approximation, which is known to accelerate primary intention healing and create optimal conditions for cell migration, whereas the adhesive forms a protective film but does not actively pull the wound edges together. Nevertheless, the difference disappeared by day 14, indicating that the adhesive does not delay final healing.

Regarding hemostasis and inflammation, our results align with those of Escobar et al. (2021), who found that cyanoacrylate adhesives significantly reduce postoperative bleeding and inflammatory signs compared with sutures [13]. The bacteriostatic properties of cyanoacrylates and the absence of foreign-body reaction from suture material likely contribute to this benefit [9,10]. Importantly, the adhesive group also required no suture removal, which is an advantage for patient comfort and reduces the need for an additional clinical visit. Patients receiving sutures had higher inflammation scores during the first few postoperative days, which is consistent with other reports [24,26,27]. The hyperemia and edema in the suture group are likely related to the mechanical trauma of the needle and thread passing through the tissues [27,28].

The collagen sponge group exhibited lower EHS values (4.0 at day 7, 6.0 at day 14), which is expected in secondary intention healing where complete epithelial edge apposition (CSR = 6) does not occur. These values should not be interpreted as “poor healing” but rather as a different healing trajectory. The collagen sponge provided a biocompatible scaffold that supported granulation tissue formation without complications, although complete re-epithelialisation required more than 14 days [14,15]. Our results are in line with those of Alvarez-Medina et al. (2023), who reported that collagen sponges are effective for palatal wound healing but require longer time compared with primary closure methods [17]. Similarly, Deng et al. (2024) found that collagen sponges were associated with slower but still satisfactory healing in oral mucosal wounds [16]. Notably, the collagen sponge was applied without sutures, and no cases of spontaneous detachment occurred, confirming that fibrin-based adherence is sufficient for clinical use, as previously shown by Schinini et al. (2021) [19]. Of note, collagen sponges create a three-dimensional structure that supports cell migration and differentiation, which may explain their moderate-to-low levels of postoperative inflammation and their potential to minimize scarring [15,29,30,31].

To our knowledge, this is the first study to directly compare sutures and cyanoacrylate adhesive using the validated EHS in a large cohort, while including a collagen sponge group for descriptive comparison (without invalid statistical claims). Previous studies typically compared only two methods (e.g., sutures vs. adhesive or sutures vs. collagen sponge) and often used heterogeneous outcome measures [20,21,32]. Our head-to-head comparison under standardized conditions provides a clearer picture of the relative merits of each technique.

Our findings support a tailored approach to palatal donor site management. When rapid epithelialization is the priority (e.g., in patients who need to resume normal diet quickly), traditional sutures remain a reliable choice. When minimizing postoperative inflammation, bleeding, and patient discomfort is the goal, cyanoacrylate adhesive was associated with better outcomes in these domains, while achieving equivalent final healing by day 14. The adhesive also eliminates the need for suture removal, which is particularly beneficial in apprehensive patients or those living far from the clinic. In this descriptive analysis, collagen sponges were associated with slower healing. They may be considered when a simple, non-invasive dressing is desired, but this observation is based on a descriptive reference group and should not be interpreted as a direct comparison with primary closure methods. The sponge’s ability to adhere spontaneously without additional fixation simplifies the procedure and reduces operative time.

When selecting a postoperative wound closure method, several limitations must be considered. Collagen sponges may not be suitable for patients with allergy to animal proteins or in cases where high tissue bond strength is required, such as in deeper or more complex wounds [33]. Surgical adhesive may be ineffective when the wound is large or has an irregular shape [34]. Thus, the choice of method should be individualized based on wound characteristics and patient needs.

Our secondary analysis revealed that the amount of granulation tissue on day 7 was significantly different between groups (highest in the adhesive group, lowest in the collagen sponge group) and was strongly correlated with final healing outcome in the primary-healing groups (ρ = 0.782). This finding is novel and clinically useful. It implies that the early inflammatory-proliferative phase, reflected by granulation tissue formation, is a key determinant of subsequent epithelialization. To our knowledge, no previous study has quantified this relationship in palatal donor sites. Future research should validate this threshold (≥2) in independent cohorts, as the present study provides the initial evidence for its predictive value.

Castro-Gaspar et al. evaluated the time to first epithelial layer formation and restoration to baseline, finding that an epithelial layer was present in nearly all patients by 21 days after surgery [24]. However, other authors have reported significantly shorter healing times in patients receiving cyanoacrylate [26]. Early epithelialization at 15 days was noted by Vastani, although those authors used smaller graft sizes [27]. Complete early epithelialisation was observed in all patients in both groups by two months. Our results, with complete early epithelialisation (EHS = 10) by day 14 in the suture and adhesive groups, compare favorably with these reports, likely due to the use of the single-incision technique and standardized postoperative care.

The ROC-derived threshold of ≥2 provides a simple binary tool that has not been previously established for palatal donor sites. With a positive predictive value of 84.9%, a patient exhibiting at least moderate granulation tissue on day 7 can be confidently expected to achieve excellent healing (EHS ≥ 9) by day 14, whereas a score of 0–1 should prompt closer follow-up or adjunctive therapy. This extends our correlation analysis by offering a clinically actionable decision rule.

The finding that larger graft area impaired healing only in the collagen sponge group is novel and clinically relevant. It suggests that primary closure methods (suture or adhesive) effectively neutralise the negative effect of wound size, whereas secondary intention healing remains sensitive to defect dimensions. For wounds exceeding approximately 2 cm^2^, primary closure should be preferred over collagen sponges. No previous study has directly compared these differential responses.

Age > 45 years and graft area > 2.0 cm^2^ independently predicted EHS ≤ 5 at day 7 in the suture group, but not in the adhesive group. This exploratory finding suggests that the adhesive may be associated with better early healing in older patients or those with larger defects, possibly due to its immediate hemostatic action and absence of suture-related tissue trauma. While these findings require prospective validation, they offer initial guidance for personalised method selection: consider adhesive instead of sutures in patients aged over 45 years or with graft areas exceeding 2 cm^2^.

Collectively, these findings support a tailored, evidence-based selection of closure method and introduce a simple prognostic tool (day-7 granulation score ≥ 2) for early identification of patients likely to achieve complete healing by day 14.

### 4.2. Strengths and Limitations

This study has several notable strengths: (i) large sample size (300 patients, 100 per group) providing adequate statistical power; (ii) standardized surgical technique (single-incision) and postoperative care; (iii) use of a validated, reliable composite index (EHS) with high inter-examiner agreement (ICC = 0.84); (iv) blinding of outcome assessors to the closure method; (v) inclusion of both primary and secondary outcomes, including a novel prognostic marker (granulation tissue); and (vi) adherence to STROBE and OHStat reporting guidelines.

The study also has limitations that must be acknowledged. First, the retrospective, non-randomized design carries an inherent risk of selection bias, as the choice of closure method was based on surgeon preference rather than random allocation. Nevertheless, three factors mitigate this concern to some extent: (i) baseline demographic and clinical characteristics (age, sex, graft area, comorbidities) were well balanced across the three groups (Table 1), with no statistically significant differences; (ii) all surgical procedures were performed by three equally experienced oral surgeons (>10 years of experience in mucogingival surgery), each of whom used all three closure methods during the study period; and (iii) the postoperative care protocol was strictly standardized. Despite these mitigating factors, residual confounding (e.g., subtle differences in surgical technique or wound characteristics not captured in our database) cannot be completely excluded. Furthermore, the clinical decision process for selecting a closure method, although systematic in practice, was not based on a predefined scoring algorithm, which limits the reproducibility of the treatment allocation in other settings. Crucially, because of the non-randomized design, none of the observed differences between groups should be interpreted as causal treatment effects. All findings are observational associations and may be influenced by unmeasured confounding (e.g., wound morphology, bleeding tendency, tissue thickness), which we acknowledge as a major limitation. Second, the study was conducted at a single center, which may limit generalizability to other settings with different patient populations or surgical protocols. Third, we did not assess patient-reported outcomes such as pain (visual analog scale) or quality of life, which are important for evaluating the clinical relevance of the differences observed. Fourth, the follow-up period was limited to 14 days for the primary outcome; complications were monitored up to day 14 per protocol; longer-term outcomes (e.g., scar quality, sensory disturbances) were not evaluated. Fifth, the EHS was originally validated for primary intention healing. Applying it to the collagen sponge group (secondary intention) introduces a systematic bias because the CSR component assumes edge-to-edge contact. Therefore, comparisons of total EHS between the collagen group and the primary closure groups are not valid measures of healing quality. We have therefore limited direct statistical comparisons to the suture and adhesive groups and report collagen group data descriptively. A validated wound score for palatal secondary intention healing is currently lacking, which is a limitation of the entire field. Sixth, we excluded smokers and patients with uncontrolled systemic diseases. It was intentional to reduce confounding, as these factors are known to impair wound healing. Consequently, our findings apply strictly to healthy non-smokers without major comorbidities. However, this limitation is shared by many published studies on palatal wound healing. Future prospective trials should specifically evaluate the performance of these closure methods in smokers and medically compromised patients, as their needs may differ.

### 4.3. Future Research Directions

Prospective randomized controlled trials are needed to confirm our findings and eliminate selection bias. Such trials should include patient-reported outcomes (pain, satisfaction, quality of life) and cost-effectiveness analyses. Longer follow-up (e.g., 3–6 months) would assess potential late complications such as scarring or altered sensation. Additionally, combining collagen sponges with bioactive agents (e.g., growth factors, platelet-rich fibrin) might accelerate healing and bring their performance closer to that of primary closure methods. Future studies should also explore hybrid approaches that combine the mechanical advantages of sutures with the biological benefits of adhesives and collagen matrices. Investigating healing time, patient comfort, and long-term results across different patient subgroups will be crucial for clinical practice. Finally, the prognostic value of early granulation tissue should be validated in an independent cohort, and a clinically useful threshold score should be confirmed.

## 5. Conclusions

This retrospective cohort study provides a comparative evaluation of three wound closure methods—polypropylene sutures, butyl-2-cyanoacrylate tissue adhesive, and a bovine collagen sponge—for palatal donor site healing after subepithelial connective tissue or free gingival graft harvesting. The key conclusions are as follows:

1. Sutures and cyanoacrylate adhesive are both effective, achieving excellent healing by day 14 (median EHS = 10), with the adhesive providing better hemostasis and less inflammation at day 7.
2. The collagen sponge results in slower healing (median EHS = 6 at day 14) in this descriptive analysis.
3. The amount of granulation tissue on day 7 is a strong prognostic marker of successful healing by day 14 (Spearman’s ρ = 0.782).
4. A day-7 granulation score ≥ 2 may predict excellent healing (EHS ≥ 9) by day 14 (sensitivity 89.4%, specificity 76.0%, AUC = 0.806); larger graft area impairs healing only in the collagen group; in suture-closed wounds, age > 45 years and graft area > 2.0 cm^2^ predict poor early response.
5. The day-7 granulation score may help identify patients who could benefit from earlier reassessment, but this finding requires prospective validation before implementation as a triage tool.
6. Choice of closure method should be individualised, balancing healing speed, inflammation, patient comfort, and wound characteristics.
7. Future prospective randomised controlled trials are needed to confirm these findings.

In summary, both sutures and cyanoacrylate adhesive achieve excellent palatal donor site healing by day 14, with the adhesive offering a patient-friendly alternative. The collagen sponge results in slower epithelialisation, as expected for secondary intention healing, but remains acceptable for selected cases where a simple, biocompatible dressing is desired. Early granulation tissue assessment provides a practical prognostic tool for patients undergoing primary closure.

## Figures and Tables

**Table 1:** Baseline characteristics of the study groups.

Characteristic	Group 1	Group 2	Group 3	*p*-Value
Age, years, mean (SD)	42.3 (12.1)	43.1 (11.8)	41.9 (12.5)	0.782 ^1^
Sex, female, n (%)	58 (58.0)	62 (62.0)	55 (55.0)	0.621 ^2^
Graft area, cm^2^, mean (SD)	1.81 (0.58)	1.78 (0.61)	1.83 (0.59)	0.852 ^1^
Current smoker, n (%)	0 (0)	0 (0)	0 (0)	–
Systemic comorbidities, n (%)	12 (12.0)	14 (14.0)	10 (10.0)	0.678 ^2^

^1^ One-way ANOVA; ^2^ χ^2^ test.

**Table 2:** Primary outcome: EHS and component scores at day 7 and day 14.

Outcome	Group 1 Median [IQR]	Group 2 Median [IQR]	Group 3 Median [IQR]	*p*-Value ^1^
Day 7				
CSR (0–6) ^2^	6.0 [3.0–6.0]	3.0 [3.0–6.0]	0.0 [0.0–3.0]	<0.001
CSH (0–2)	1.0 [1.0–1.75]	2.0 [2.0–2.0]	2.0 [1.0–2.0]	0.006
CSI (0–2)	1.0 [1.0–1.0]	2.0 [2.0–2.0]	2.0 [1.0–2.0]	<0.001
EHS (0–10)	7.0 [5.0–9.0]	7.0 [7.0–9.0]	4.0 [3.0–5.0]	-
Day 14				
CSR (0–6) ^2^	6.0 [6.0–6.0]	6.0 [6.0–6.0]	3.0 [3.0–3.0]	<0.001
CSH (0–2)	2.0 [2.0–2.0]	2.0 [2.0–2.0]	2.0 [2.0–2.0]	0.78
CSI (0–2)	2.0 [2.0–2.0]	2.0 [2.0–2.0]	2.0 [2.0–2.0]	0.86
EHS (0–10)	10.0 [9.0–10.0]	10.0 [9.0–10.0]	6.0 [5.0–7.0]	-

^1^ For CSR, CSH, CSI: Kruskal–Wallis test with post hoc pairwise comparisons (Bonferroni correction, *p* < 0.0167 considered significant). For total EHS, no statistical comparison was performed involving the collagen group; *p*-values are therefore not reported (indicated by “-”). Comparisons between suture and adhesive groups are reported in the text (Mann–Whitney U test, *p* = 0.31 at day 7, *p* = 0.82 at day 14). Data are median [interquartile range] unless otherwise stated. ^2^ CSR scores in the collagen group represent coverage of the defect with granulation tissue and migrating epithelium, not edge-to-edge approximation as in primary healing. Total EHS values reflect secondary intention healing and are not directly comparable to primary healing groups. Statistical comparisons between suture and adhesive are reported in the text.

**Table 3:** Amount of granulation tissue at day 7 (secondary outcome).

Score ^1^	Group 1 n (%)	Group 2 n (%)	Group 3 n (%)
0 (absent/minimal)	2 (2)	0 (0)	8 (8)
1 (small area)	18 (18)	5 (5)	72 (72)
2 (moderate)	68 (68)	15 (15)	18 (18)
3 (abundant)	12 (12)	80 (80)	2 (2)
Median [IQR]	2.0 [2.0–3.0]	3.0 [2.0–3.0]	1.0 [1.0–2.0]

^1^ Kruskal–Wallis test: *p* < 0.001 for overall comparison. Post hoc pairwise comparisons with Bonferroni correction: adhesive vs. sutures *p* = 0.024; adhesive vs. collagen *p* < 0.001; sutures vs. collagen *p* < 0.001.

**Table 4:** Analysis of poor early responders in the suture group.

Variable	Poor Responders (*n* = 15)	Good Responders (*n* = 85)	*p*-Value
Age, years (mean ± SD)	50.1 ± 10.8	41.2 ± 11.9	0.008
Graft area, cm^2^ (mean ± SD)	2.28 ± 0.52	1.75 ± 0.49	<0.001
Female sex, n (%)	8 (53%)	50 (59%)	0.67
Surgeon (A/B/C) distribution	5/5/5	29/28/28	0.99

## Data Availability

The datasets generated and analyzed during the current study are not publicly available due to patient privacy and institutional data protection policies. An anonymized minimal dataset is available from the corresponding author upon reasonable request and after approval by the Institutional Ethics Committee.

## Abbreviations

The following abbreviations are used in this manuscript:

EHS	Early Wound Healing Score
IQR	interquartile range
ROC	receiver operating characteristic
AUC	area under the curve
FGG	free gingival graft
CTG	connective tissue graft
ICC	intraclass correlation coefficient
CI	confidence interval
STROBE	Strengthening the Reporting of Observational Studies in Epidemiology
OHStat	Oral Health Statistics
ICMJE	International Committee of Medical Journal Editors
CLIP	Clinical Images and Photographs
HbA1c	glycated hemoglobin
CSR	Clinical Signs of Re-epithelialization
CSH	Clinical Signs of Hemostasis
CSI	Clinical Signs of Inflammation
SD	standard deviation
ANOVA	analysis of variance
PSM	propensity score matching
SMD	standardized mean differences
OR	odds ratio
AIC	Akaike’s Information Criterion
VIF	variance inflation factor
NPV	negative predictive value
